# Natural History, Complications and Long-Term Prognosis of Patients with Cardiac Ascites

**DOI:** 10.3390/jcm15218537

**Published:** 2026-11-02

**Authors:** Moran Ben Yosef Lavi, Shaul Yaari, Zvi Ackerman

**Affiliations:** Department of Medicine, Hadassah-Hebrew University Medical Center, Mount Scopus Campus and the Hebrew University-Hadassah Medical School, Jerusalem 91240, Israel; moranbenyosef@gmail.com (M.B.Y.L.); yaari@hadassah.org.il (S.Y.)

**Keywords:** metabolic dysfunction-associated steatotic liver disease (MASLD), cardiac ascites, serum and ascitic fluid total protein and albumin, all-cause mortality, survival time, Fibrosis-4 Index (FIB-4)

## Abstract

**Introduction and Objectives:** The natural history of patients with cardiac ascites (CA) due to congestive heart failure (CHF) remains poorly documented. This study aims were to characterize the natural history of patients with CA including its complications and long-term prognosis, in comparison to patients with ascites secondary to metabolic dysfunction-associated steatotic liver disease (MASLD). **Patients and Methods:** We conducted a retrospective evaluation of 98 patients presenting with ascites due to either CHF (N = 51) or MASLD (N = 47). **Results:** Patients with CHF exhibited a higher prevalence of cardiac-specific pathology compared to patients with MASLD. Throughout the study period, both groups presented a decline in serum and ascitic total protein and albumin levels. In patients with CHF, the decline in ascitic total protein was so substantial that levels dropped below 25 g/L in 16 patients. Furthermore, Fibrosis-4 Index (FIB-4) values increased during the study, with a more significant rise observed in the patients with MASLD. Patients with CHF had a significantly longer mean survival time compared to those with MASLD (25.0 ± 3.5 vs. 17.0 ± 2.1 months, *p* = 0.041). Independent predictors of shorter survival included a higher monthly frequency of paracentesis and rising FIB-4 scores during the study period. Conversely, higher admission levels of ascitic fluid albumin were associated with improved survival. **Conclusions:** Patients with CA demonstrate better survival outcomes than those with ascites secondary to MASLD. As cardiac disease progresses, serum and ascitic albumin and total protein levels typically decline. These findings suggest that the utility of ascitic fluid analysis in the differential diagnosis of ascites may be limited in cases of advanced CHF.

## 1. Introduction

In recent years, there has been a significant increase in the global prevalence of congestive heart failure (CHF) [1]. This rise is attributed to an aging population and the increasing availability of effective, evidence-based therapies that prolong life in patients with CHF [1]. The development of ascites in patients with CHF is an infrequent clinical event; CHF is identified as the underlying etiology in approximately 3% of patients admitted for the evaluation of ascites [2,3]. While ascites in patients with liver cirrhosis is primarily associated with increased portal pressure, the pathophysiology of cardiac ascites (CA) is distinct. Decompensated right ventricular failure leads to elevated right atrial pressure, which is transmitted via the inferior vena cava and hepatic veins to the liver sinusoids. The resulting sinusoidal congestion and enlarged fenestrae lead to the exudation of protein-rich fluid into the space of Disse. Initially, this fluid is drained by the hepatic lymphatics; however, once the volume of exudate overwhelms the lymphatic system’s capacity, it shunts into the peritoneal cavity and manifests clinically as CA [4].

The appearance of ascites in a cirrhotic patient is an unfavorable prognostic marker, significantly decreasing the probability of long-term survival [5,6,7,8]. This diminished survival is due to deteriorating liver function and the frequent occurrence of complications, such as spontaneous bacterial peritonitis (SBP), hepatorenal syndrome-acute kidney injury (HRS-AKI), hepatic encephalopathy (HE), and bleeding from esophageal or gastric varices [5,6,7,8].

Over the last few decades, a significant increase in the prevalence of obesity, metabolic dysfunction-associated steatotic liver disease (MASLD), and metabolic dysfunction-associated steatohepatitis (MASH) has been observed in the general population [9,10]. Patients with MASLD or MASH are predisposed not only to the complications of cirrhosis and portal hypertension but also to various cardiac pathologies, including cirrhotic cardiomyopathy, ischemic heart disease, and CHF [11,12,13,14]. The development of ascites in a patient with MASLD-related cirrhosis presents a significant diagnostic and therapeutic challenge. It may arise as a complication of hepatic pathology, cardiac pathology, or the synergistic effect of both conditions [11,12,13,14,15]. In contrast to cirrhotic patients, the natural history, longitudinal complications, and long-term prognosis of patients with CA remain poorly documented [3,4]. The primary objectives of this study were to characterize the features of ascites, its complications, and the long-term prognosis of patients with chronic CHF in comparison to patients with ascites secondary to MASLD-related cirrhosis.

## 2. Patients and Methods

### 2.1. Setting and Population

This retrospective historical cohort study included patients with either chronic CHF or MASLD-related cirrhosis who were admitted to either of the two Hadassah Medical Organization (HMO) campuses in Jerusalem between November 2005 and May 2021 (15.5 years) for the evaluation of ascites. MASLD was diagnosed by the recently published criteria of European Association for the Study of the Liver [10].

### 2.2. Inclusion and Exclusion Criteria

All included patients demonstrated “high-gradient ascites” during their initial paracentesis, defined by a serum-ascites albumin gradient (SAAG) ≥11 g /L [2]. Patients with an ascitic total protein (TP) of ≥25 g/L were categorized as having CA, while those with ascitic TP <25 g/L were categorized as having ascites due to their liver disease [4].

Eligibility was limited to patients who underwent ≥3 therapeutic paracentesis sessions at least one week apart, had at least two ascitic fluid samples submitted for biochemical analysis and had at least one ascitic sample submitted for microbial culture. Exclusion criteria included HIV infection; a history of active or previous malignancy; chronic peritoneal dialysis; or any peritoneal pathology potentially responsible for ascites. Furthermore, patients with non-MASLD liver diseases were excluded, including active or past HCV/HBV infection, hemochromatosis, Wilson’s disease, Budd–Chiari syndrome, autoimmune hepatitis, primary biliary cholangitis, sarcoidosis, alcoholic liver disease, or chronic toxic liver injury.

### 2.3. Data Collection and Outcomes

Data collection for our study began in the summer of 2021. Clinical and laboratory data were collected from medical records documented during periodic day-care paracentesis visits and hospitalizations for acute deterioration. Follow-up data were collected for each patient from inclusion (first paracentesis) until death or the final recorded paracentesis (for patients alive in May 2021).

For patients with MASLD-related cirrhosis who underwent liver transplantation during the follow-up period, the date of transplantation was recorded as the primary endpoint (censored as death), regardless of their post-transplantation clinical course.

### 2.4. Echocardiographic Assessment

Reports of echocardiographic assessments performed during the study period were reviewed. The following parameters were extracted from the formal reports: left and right ventricular function, the presence and severity of pulmonary hypertension, and the degree of mitral and tricuspid regurgitation. These echocardiograms were performed and interpreted by attending cardiologists at the respective medical centers. The decision to order an echocardiographic examination was made independently by the attending physicians responsible for patient care; notably, no research was envisioned at the time these clinical assessments were performed.

### 2.5. Statistical Analysis

Descriptive statistics include percent for categorical data and mean and standard deviation for continuous variables. Differences between categorical variables were assessed by Chi-square test or Fisher’s exact test, when necessary. Comparison between 2 independent groups was performed using Mann–Whitney U test and between 4 independent groups using Kruskal–Wallis test. In order to investigate the effect of categorical variables on the overall survival, comparative analysis using the Kaplan–Meier method compared by the log-rank test was performed. In order to investigate the variables that were associated with long term survival, Cox regression analysis was used. Variables that were found to be statistically significant and were considered clinically relevant at univariate analysis were entered in multivariate logistic regression analysis. A *p*-value of <0.05 was considered to be statistically significant. The Statistical Package for Social Sciences (SPSS) version 23 (IBM Corp., Armonk, NY, USA) was used to analyze the data.

### 2.6. Ethical Considerations

The research proposal was approved by the Helsinki Committee of the HMO (No: 0308-21-HMO).

## 3. Results

### 3.1. Patient Characteristics

This study included 98 patients with ascites. Based on ascitic fluid analysis, 51 patients were categorized as having CA and 47 as having ascites secondary to their liver disease. More than half of the total cohort was male. The mean age at presentation and the mean total number of paracentesis sessions performed during the study period were similar between both groups (Table 1). However, MASLD patients required a higher frequency of paracentesis sessions per month and were significantly more likely to receive intravenous albumin infusions following these procedures (Table 1). These clinical decisions regarding paracentesis and albumin administration were made independently by attending physicians as part of standard care, without prior research intent.

### 3.2. Clinical and Diagnostic Findings

Patients with CHF demonstrated a significantly higher prevalence of ischemic heart disease and chronic atrial fibrillation. Conversely, patients with MASLD underwent a greater number of liver biopsies and exhibited higher rates of esophageal varices, HE and splenomegaly (Table 1).

Echocardiographic reports of ninety-seven patients (50 patients from the CHF group and 47 from the MASLD) were available for our review. Echocardiographic findings in patients with CHF revealed higher rates of valvular, non-valvular, and myocardial pathologies, as well as pulmonary hypertension, compared to patients with MASLD (Table 2).

### 3.3. Overlapping Pathologies and Clinical Profiles

Within the CA cohort (n = 51), 11 patients (21.6%) presented with clinical and laboratory findings consistent with advanced MASLD, in addition to their primary cardiac disease (Table S1). Similarly, in the MASLD group (n = 47), 11 patients (23.4%) exhibited findings compatible with underlying cardiac pathology alongside their liver disease (Table 1, Table 2, Tables S1 and S2).

### 3.4. Baseline Biochemical and Hematological Comparisons

Significant differences in baseline laboratory parameters were observed between the two groups. Upon inclusion, patients with CHF demonstrated significantly higher serum levels of albumin, urea, and creatinine, as well as elevated white blood cell and platelet counts compared to the MASLD group. Conversely, serum levels of alanine aminotransferase (ALT), aspartate aminotransferase (AST), and bilirubin were significantly lower in the CHF group (Table 3 and Table S3).

### 3.5. Dynamic Biochemical Trends

Throughout the follow-up period, dynamic changes in serum and ascitic biochemical parameters were observed in both cohorts. In the serum, a more prominent increase in creatinine levels was noted in the MASLD group compared to the CHF group. While both groups experienced a comparable decline in serum TP, the reduction in serum albumin levels was significantly more pronounced in patients with CHF (Table 3 and Table S3). Significant shifts were also observed in the ascitic fluid analysis. A decline in ascitic albumin levels occurred at a similar rate in both groups; however, the decline in ascitic TP was markedly higher in the CHF group. This reduction was so substantial that 16 patients in the CHF group eventually exhibited ascitic TP levels below 25 g/L (Table 4 and Table S4). This is clinically significant, as 25 g/L is the traditionally recognized diagnostic threshold for identifying cardiac etiology in high-gradient ascites [2,4].

### 3.6. Infectious Complications

During the follow-up period, 10 clinical episodes of SBP occurred in 10 unique patients across both groups. This included six patients with MASLD and four with CHF (*p* = NS, Fisher’s exact test). All patients diagnosed with SBP met the standardized clinical and laboratory criteria: a suggestive clinical presentation, an ascitic fluid neutrophil count >250 cells/mm^3^, and a positive bacterial culture from either blood or ascitic fluid [15].

### 3.7. Liver Fibrosis Progression

The Fibrosis-4 (FIB-4) index, a non-invasive estimate of liver fibrosis, was calculated for each participant at study inclusion and at the conclusion of the study period [16]. While both groups exhibited an increase in FIB-4 values over time, this progression was significantly more prominent in patients with MASLD (Table 3 and Table S3).

### 3.8. Mortality and Survival Outcomes

By the end of the study period, 92 patients had died, including nine MASLD patients who underwent liver transplantation. As previously defined, the date of transplantation was recorded as the primary endpoint (censored as death) for these individuals. At the conclusion of the study, four patients with CHF and two patients with MASLD remained alive.

Mortality data were obtained from the Ministry of Interior records. These records provided the date of death but lacked specific medical details regarding the cause of death. Furthermore, as many participants received outpatient care at various community medical facilities, reliable information concerning the exact cause of death could not be obtained.

### 3.9. Survival Analysis

To compare overall survival between the two groups, a Kaplan–Meier curve was constructed (Figure 1). Due to the unavailability of specific causes of death, all-cause mortality was utilized for this analysis. The mean survival time was significantly higher in patients with CHF compared to those with MASLD [25.0 ± 3.5 (95% CI, 18.1–31.9), vs. 17.0 ± 2.1 (95% CI 12.9–21.2) months, log-rank test, *p* = 0.041].

### 3.10. Predictors of Mortality

Univariate analyses were performed to identify parameters associated with survival within the study population. The following variables were found to have no significant effect on overall survival: demographics & habits (age, gender, and active smoking status), comorbidities (hypertension, hyperlipidemia, diabetes mellitus, chronic lung disease, hypothyroidism, or a history of cerebrovascular accident), cardiac findings (left or right ventricular dysfunction, ischemic heart disease, chronic atrial fibrillation, pulmonary hypertension, and mitral or tricuspid valve pathology), hepatic & clinical indicators (presence of esophageal varices, splenomegaly, or HE), and interventions (like treatment with anticoagulants or a history of albumin infusion during paracentesis sessions).

### 3.11. Univariate Survival Analysis

A second analysis was performed using Cox proportional hazards regression to evaluate the impact of continuous variables on overall survival. Univariate analysis identified several parameters significantly associated with decreased survival: clinical interventions (a higher mean monthly frequency of paracentesis sessions), renal and hepatic function (higher admission serum levels of urea, creatinine, AST, ALT, and total bilirubin), hematological and fibrosis markers [elevated mean corpuscular volume (MCV), higher final FIB-4 values, and a greater percentage increase in FIB-4 during the follow-up period]. Conversely, higher baseline levels of serum albumin, as well as higher ascitic fluid total protein and albumin at admission, were significantly associated with improved survival outcomes (Table 5).

### 3.12. Multivariate Survival Analysis

Following the univariate analysis, three multivariate Cox proportional hazards models were constructed to identify independent predictors of mortality (Table 6). In the **first model**, which included all variables associated with survival in the univariate analysis, the following factors were found to be independently associated with decreased survival: Monthly frequency of paracentesis sessions during the follow-up period, admission serum AST levels and MCV values and the percentage change in FIB-4 values observed during the study period (Table 6).

Two additional Cox proportional hazards models were constructed to further delineate the independent predictors of mortality (Table 6). **Model 2:** After excluding the frequency of paracentesis sessions from the analysis, the variables independently associated with decreased survival were admission serum urea, AST levels, and MCV values, alongside the final FIB-4 values recorded at the last follow-up visit. Conversely, higher admission ascitic fluid albumin levels remained independently associated with improved survival. **Model 3:** When the analysis was restricted to variables recorded only at the time of study inclusion, admission serum urea and AST levels were identified as independent predictors of decreased survival. Higher admission ascitic fluid albumin levels continued to be a significant predictor of improved survival (Table 6).

## 4. Discussion

This retrospective cohort study included 98 patients admitted over a 15.5-year period for the evaluation of ascites. Our primary objective was to characterize the clinical features, complications, natural history, and long-term prognosis of patients with CA, utilizing patients with ascites secondary to MASLD-related cirrhosis as a comparative cohort.

Upon stratification, we observed significant overlap between these two conditions: 11 patients (21.6%) in the CA group also presented with MASLD-induced liver disease, while 11 patients (23.4%) in the MASLD group exhibited concomitant cardiac pathology. The complex interplay and reciprocal interactions between hepatic and cardiac pathologies are well-recognized in clinical literature [11,12,13,14,17].

### 4.1. Cardiovascular Involvement in Cirrhotic Patients

Patients with cirrhosis of various etiologies, even in the absence of pre-existing cardiac disease, may develop cirrhotic cardiomyopathy [14]. This clinical entity is characterized histologically by cardiac hypertrophy, patchy fibrosis, and subendothelial edema. Physiologically, it manifests as impaired contractile responsiveness to stress, diastolic dysfunction, and electrophysiological abnormalities [14].

### 4.2. Cardiovascular Involvement in MASLD Cirrhotic Patients

MASLD cirrhosis, much like liver diseases of other etiologies, is a multisystemic condition that affects extrahepatic organs, including the heart and the peripheral vasculature [17]. The presence of MASLD cirrhosis may accelerate coronary atherosclerosis and induce myocardial abnormalities, such as cardiac remodeling, hypertrophy, and arrhythmias (e.g., chronic atrial fibrillation). These changes often precede and promote the development of new-onset CHF [11].

The increased incidence of cardiovascular events and CHF in patients with MASLD cirrhosis remains independent of a broad range of confounding risk factors, including age, sex, ethnicity, and adiposity measures and other additional factors that are present in patients with MASLD like insulin resistance, diabetes mellitus hypertension, or other common cardiovascular risk factors. Furthermore, the risk appears to correlate with the severity of MASLD, particularly in patients with advanced fibrosis [11,17].

Epidemiological studies reveal that in subjects with MASLD, cardiovascular disease is documented as one of the leading causes of death, whereas liver-related complications account for approximately 10% of deaths [18].

### 4.3. Hepatic Involvement in Patients with CHF

Hepatic involvement is common in patients with right-sided CHF of any etiology [19,20]. These patients may present with histopathological changes in the hepatic parenchyma, including sinusoidal dilatation, centrizonal hepatocyte atrophy, necrosis, and centrizonal fibrosis. In advanced stages of right-sided heart failure, portal fibrosis may also be observed [19,20]. The degree of congestive hepatic fibrosis correlates with several key parameters: right atrial pressure, right atrial diameter and right ventricular function impairment. As previously noted, advanced liver fibrosis triggers specific pathophysiological mechanisms that further impair cardiac anatomy and function. These include the impairment of natriuresis, which increases cardiac preload, worsening ventricular hypertrophy, and a progression from left ventricular diastolic dysfunction to systolic dysfunction and eventual right ventricular failure [19,20].

### 4.4. Cardiovascular and Renal Effects of Paracentesis

Patients in both groups underwent periodic percutaneous paracentesis on an as-needed basis, primarily for symptomatic relief. The frequency of paracentesis sessions (per month) and the proportion of patients receiving post-paracentesis albumin infusion were significantly higher in the patients with MASLD.

The use of repeated paracentesis for symptomatic relief in patients with CA has been documented in several studies [21,22,23]. Kunin et al. observed that patients undergoing regular, scheduled paracentesis (rather than on an “as-needed” basis) over a period exceeding three months showed significant clinical improvement. Specifically, these patients demonstrated an upgraded New York Heart Association functional class and a reduction in serum creatinine, C-reactive protein, and Brain Natriuretic Peptide (BNP) levels [21].

It has been suggested that the cardiac and renal improvements observed following regular paracentesis may be attributed to several physiological mechanisms: mechanical removal of the ascites fluid, reduction in intraabdominal pressure, improved renal perfusion following the reduction of pressure on the renal veins, increase in venous return to the right heart, increased right ventricular end diastolic volume and improved right ventricular stroke volume. Moreover, removal of ascites fluid may also cause decompression of the descending aorta that may be followed by decreased left ventricular afterload [21,22,23].

### 4.5. Dynamics of Serum and Ascitic TP and Albumin During the Study Period

During the study period, a decline in serum and ascitic fluid TP and albumin levels was observed in both groups. However, among patients with CA, the decrease in ascitic TP levels was particularly pronounced; in 16 patients, levels fell below 25 g/L. This is notable as 25 g/L is generally considered the minimum threshold for ascitic fluid TP in patients with CHF [2,3]. Similar findings have been previously reported in cases of advanced CHF [24].

The decrease in serum and ascitic fluid TP and albumin levels in patients with CHF may be driven by several pathophysiological mechanisms: (a) Chronic Inflammation: CHF is associated with a chronic inflammatory state that exerts a catabolic effect. This can lead to anorexia, reducing nutritional intake while simultaneously increasing resting energy expenditure and inhibiting hepatic synthesis of albumin and protein [25]. (b) Hepatic Sinusoidal Remodeling: A 48% increase in FIB-4 values was observed among CA patients during our study. This progression of hepatic fibrosis likely results from a sequence of events: worsening right-sided heart failure leads to chronic passive congestion, increased collagen deposition in the Space of Disse, and “capillarization” of the hepatic sinusoids. As the number of functioning fenestrae decreases, the sinusoids become less permeable (“leaky”), significantly reducing the leakage of TP and albumin into the ascitic fluid [25,26]. (c) Gastrointestinal congestion: Venous congestion in the intestines can cause early satiety and nausea, leading to decreased food intake and promoting enteric protein-losing enteropathy [25]. (d) Dilutional hypoalbuminemia: Elevated systemic venous pressure promotes the leakage of albumin into the extravascular space, contributing to dilutional hypoalbuminemia [25]. (e) Albuminuria: Protein loss via the urine, particularly observed in adult patients with congenital heart disease [24].

The decline in ascitic TP and albumin levels in these patients likely results in diminished opsonic activity within the fluid. This biochemical shift may serve as a trigger for the development of SBP episodes in this population [27].

The decreasing reliability of ascitic fluid TP as a diagnostic tool in patients with advanced, chronic CHF, particularly those with concurrent hepatic pathology, presents a significant clinical challenge. Relying solely on TP in these complex cases can be misleading, necessitating the use of supplemental diagnostic modalities to accurately differentiate the etiology of the ascites [28].

### 4.6. Survival Outcome

At the end of the study period, the mean survival time for patients with CA was significantly higher than that of patients with MASLD (25.0 ± 3.5 vs. 17.0 ± 2.1 months, *p* = 0.041).

Similar findings have been reported in the medical literature. Kunin et al. observed that the median survival of patients with CA treated with abdominal paracentesis was 13.5 months (range: 0–34 months). Notably, a subgroup of CA patients with sonographic evidence of liver cirrhosis (characterized by a lobular liver edge, hepatic atrophy, nodular texture, and hypertrophy of the left and caudate lobes) exhibited significantly shorter survival (7.0 ± 5.6 months compared to 18.7 ± 11.2 months in the non-cirrhotic patients. *p* = 0.0212), [21]. Furthermore, a meta-analysis by Qiu et al. of six observational studies involving 12,374 patients with CHF found that those with concurrent MASLD faced a significantly higher risk of all-cause mortality, major adverse composite outcomes, and hospitalization [29].

### 4.7. Predictors of All-Cause Mortality in the Study Cohort

Patients with MASLD-related cirrhosis generally have a poor prognosis, with mortality driven primarily by cardiovascular causes and liver-related causes [18,30,31,32]. This unfavorable outcome is likely secondary to systemic metabolic dysfunction, including obesity, diabetes mellitus, hypertension, and hyperlipidemia, alongside multi-organ involvement affecting the renal, neurological, cardiovascular, pulmonary, gastrointestinal, and endocrine systems [18,30,31,32].

To identify parameters associated with poor survival in our patient population, we constructed three multivariate Cox proportional hazards models. The variables independently associated with worse survival included the monthly number of sessions performed during the study period, admission levels of serum urea and AST, MCV value, the FIB-4 value at the final follow-up visit and the percentage change in FIB-4 observed during the study period. Conversely, higher admission ascitic fluid albumin levels were associated with improved survival.

The requirement for more frequent paracentesis in patients with either CA or ascites secondary to MASLD cirrhosis may indicate that these individuals are becoming refractory to loop diuretics and vasoactive therapies. This transition often reflects a worsening of the underlying liver disease that is characterized by increasing portal hypertension, and/or a deterioration of cardiac function, which further impairs renal perfusion [5,21,22,23]. Studies have consistently shown that patients with refractory ascites of either etiology face a poor prognosis [5,21,22,23].

In our study, admission serum urea levels were an independent predictor of mortality. Similar findings have been reported in patients with acute-on-chronic liver failure and AKI [33], hospitalized patients with decompensated cirrhosis [34], and those with CHF [35].

Notably, in cirrhotic populations, serum creatinine is often less reliable for estimating renal function or predicting survival. This inaccuracy stems from several factors, including low protein intake, sarcopenia, reduced hepatic creatine synthesis and expanded volume of distribution. These factors can artificially lower serum creatinine levels, masking significant renal impairment [33,36].

### 4.8. The Predictive Role of the FIB-4 Index in the Study Cohort

As anticipated, the degree of hepatic fibrosis represented by FIB-4 values was high in both patient groups at admission and during the final follow-up visit of the study. However, FIB-4 values were significantly higher in patients with MASLD compared to those with CA at both time points (e.g., at admission and during the last follow-up visit). Furthermore, the progression of fibrosis throughout the study was more pronounced in the MASLD patients. Despite the disparity in fibrosis progression between the two groups, multivariate analysis revealed that the percentage change in FIB-4 was independently associated with worse survival in both populations.

In recent years, several reports have highlighted the prognostic significance of the FIB-4 index in cardiac populations [37,38,39]. These studies consistently conclude that elevated FIB-4 values are associated with an increased risk of cardiovascular events, cardiovascular mortality, and all-cause mortality [37,38,39]. Furthermore, baseline FIB-4 values have been identified as independent predictors of major adverse cardiovascular events (MACE) in patients with MASLD [13,40].

Longitudinal, serial FIB-4 measurements have proven effective in predicting both hepatic and cardiac outcomes in large cohorts of MASLD patients. These studies confirm that dynamic changes in the FIB-4 index are a reliable marker for monitoring liver fibrosis progression; specifically, transitioning from a low-risk to a high-risk score is associated with major advanced liver outcomes, MACE, and a higher risk of all-cause mortality [41,42].

### 4.9. Study Limitations

Our study has several limitations. First, its retrospective observational design introduces inherent selection bias. However, we believe that our selection process helped to mitigate this limitation. Second, this was a single-center study with a relatively small sample size; consequently, the findings may not be generalizable to populations with differing liver disease etiologies. Third, the study design precluded the determination of the exact cause of mortality for the included patients. Finally, N-terminal pro-BNP levels were measured in only a minority of the cohort; as a result, these data were excluded from the final analysis. Despite these limitations, our study possesses notable strengths. All laboratory testing was performed at a single facility using consistent kits and techniques, and data were retrieved directly from institutional archives rather than multiple external sources. Furthermore, patients in both groups were followed for a relatively prolonged period. This longitudinal approach allowed us to observe the natural history of patients with MASLD-related and/or cardiac-related ascites. It also enabled us to track the dynamics of various hematological and biochemical parameters in the serum, blood, and ascitic fluid, providing insights into diagnosing cardiac ascites in advanced stages when Runyon’s criteria may be less effective [28].

Our findings warrant validation in a multi-center prospective study. Future research should specifically investigate which biomarkers, diagnostic tools, and imaging modalities optimize differential diagnosis, particularly for patients presenting with concomitant cardiac and hepatic pathologies and low TP levels in the ascitic fluid.

## 5. Conclusions

Patients with CHF and ascites often present with concurrent MASLD of varying severity. Conversely, those with ascites due to MASLD may also suffer from advanced cardiac pathology. While patients with CA generally demonstrate better survival rates than those with ascites due to MASLD, the prognosis shifts as cardiac disease progresses. Increased severity of CHF can lead to worsening liver fibrosis and a concomitant decline in serum and ascitic TP and albumin levels. These biochemical shifts may predispose CA patients to complications such as SBP. Furthermore, the reduction in ascitic TP and albumin may limit the utility of fluid analysis in the differential diagnosis of ascites in patients with advanced heart failure.

## Figures and Tables

**Figure 1:**
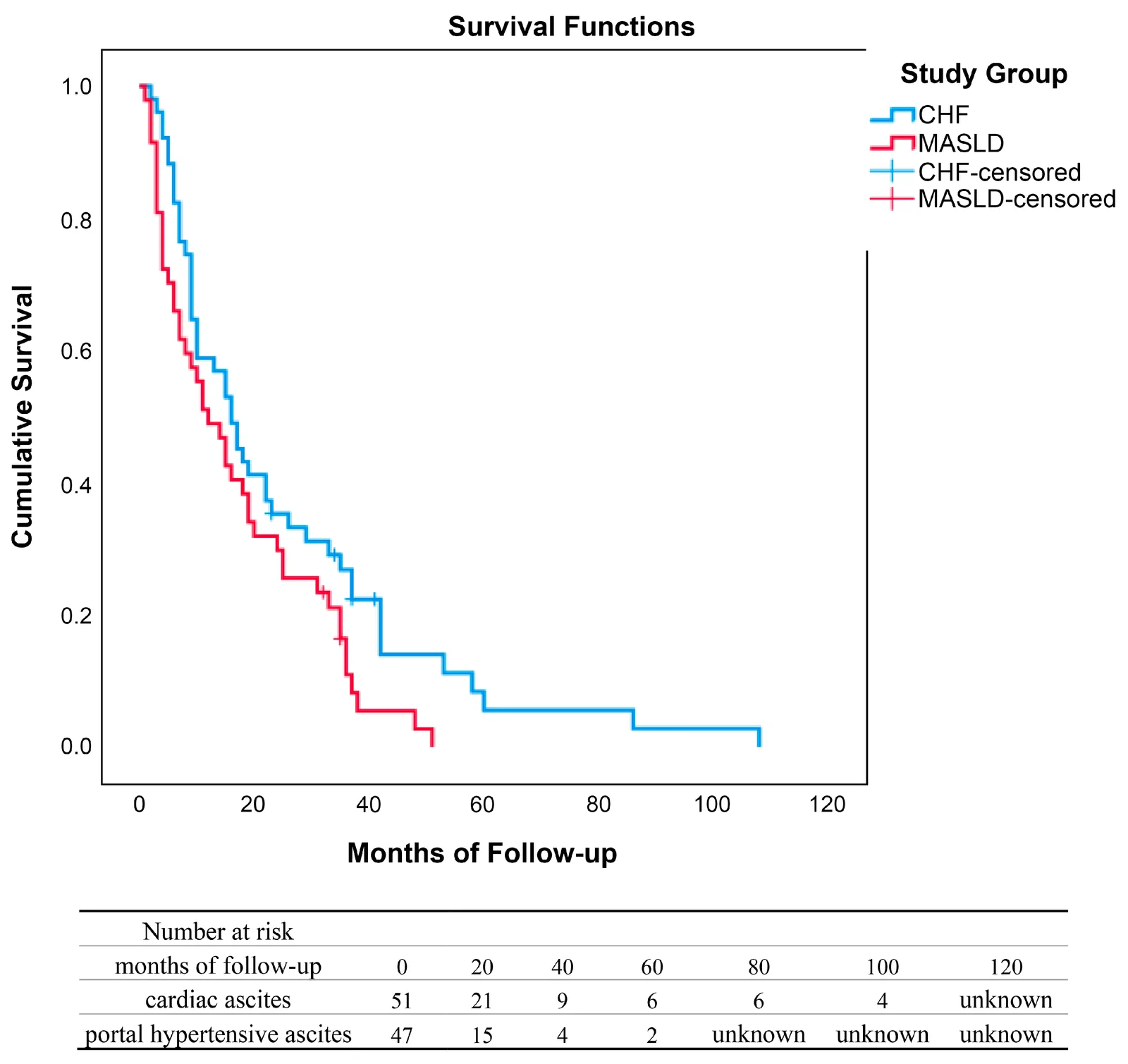
Kaplan–Meier curves for all-cause mortality in both groups of patients. Mean survival (±SD) for cardiac ascites patients was 25.0 ± 3.53 months (95% CI; 18.08–31) and for portal hypertensive ascites patients 17.02 ± 2.12 months (95% CI;12.87–21.17), Log Rank test (Mantel Cox) (*p* = 0.041).

**Table 1:** Baseline clinical characteristics of the major study groups.

Variable	CHF n = 51	MASLD n = 47	*p*-Value
**Demographics**			
Male gender	30 (58.8)	28 (59.6)	0.940
Age at presentation (years)	66.6 ± 13.4	67.9 ± 10.7	0.946
Follow-up period (months)	23.2 ± 21.5	16.7 ± 14.0	0.092
Number of paracenteses during F/U	9.0 ± 8.10	9.3 ± 7.9	0.506
Number of paracenteses per month of F/U	0.63 ± 0.62	1.02 ± 0.88	**0.028**
**Associated medical conditions**			
Diabetes Mellitus	29 (56.9)	32 (68.1)	0.252
Hypothyroidism	17 (33.3)	10 (21.3)	0.182
Chronic lung disease	14 (27.5)	14 (29.8)	0.798
Hypertension	46 (90.2)	39 (83.0)	0.293
Hyperlipidemia	46 (90.2)	39 (83.0)	0.293
Ischemic heart disease	33 (64.7)	18 (38.3)	**0.009**
Chronic atrial fibrillation	32 (62.5)	12 (25.5)	**<0.001**
Chronic anti-coagulation therapy	29 (56.9)	12 (25.5)	**0.002**
S/P CVA	10 (19.6)	7 (14.9)	0.538
Active smoking	13 (25.5)	9 (19.1)	0.452
Underwent a liver biopsy	3 (5.9)	19 (40.4)	**<0.001**
Presence of esophageal varices	4 (8.0)	26 (55.3)	**<0.001**
Presence of hepatic encephalopathy	6 (11.8)	22 (46.8)	**<0.001**
Presence of splenomegaly	10 (20.0)	26 (55.3)	**<0.001**
Albumin infusion during paracentesis	9 (17.6)	18 (38.3)	**0.022**

Parameters are presented either as mean ± standard deviation or as n (%). CHF, congestive heart failure; MASLD, Metabolic Dysfunction-Associated Steatotic Liver Disease; F/U, follow-up.

**Table 2:** Echocardiogram findings in the major study groups.

Variable	CHF	MASLD	*p*-Value
**Mitral regurgitation**	n = 47	n = 45	**<0.001**
No finding	9 (19.1)	28 (62.2)	
Mild	9 (19.1)	9 (20.0)	
Moderate	17 (36.2)	5 (11.1)	
Severe	12 (25.5)	3 (6.7)	
**Tricuspid regurgitation**	n = 46	n = 46	**<0.001**
No finding	7 (15.2)	24 (52.2)	
Mild	7 (15.2)	14 (30.4)	
Moderate	11 (23.9)	4 (8.7)	
Severe	21 (45.7)	4 (8.7)	
**Pulmonary hypertension**	n = 44	n = 47	**<0.001**
No finding	10 (22.7)	37 (78.7)	
Mild	6 (13.6)	3 (6.4)	
Moderate	13 (29.5)	4 (8.5)	
Severe	15 (34.1)	3 (6.4)	
**Left Ventricular Dysfunction**	n = 50	n = 47	**<0.001**
No finding	7 (14.0)	36 (76.6)	
Mild	11 (22.0)	6 (12.8)	
Moderate	15 (30.0)	2 (4.3)	
Severe	17 (34.0)	3 (6.4)	
**Right Ventricular Dysfunction**	n = 48	n = 43	**<0.001**
No finding	7 (14.6)	36 (83.7)	
Mild	7 (14.6)	5 (11.6)	
Moderate	16 (33.3)	1 (2.3)	
Severe	18 (37.5)	1 (2.3)	

Parameters are presented in number (%) of patients that underwent echocardiography examination and the result of the parameter examined is reported. CHF, congestive heart failure; MASLD, Metabolic Dysfunction-Associated Steatotic Liver Disease.

**Table 3:** Blood hematological and biochemical parameters of the major study groups at admission (A) and at the last (L) follow-up visit.

Variable	CHF n = 51	MASLD n = 47	*p*-Value
Urea (A), mmol/L	17.7 ± 9.6	9.6 ± 7.5	**<0.001**
Creatinine (A), µmol/L	193 ± 126	124 ± 106	**<0.001**
Creatinine (L), (µmol/L)	269 ± 149	264 ± 137	0.934
% Change in creatinine	(+)70 ±110	(+)177 ±155	**<0.001**
Total protein (A), g/L	67.6 ± 11.0	66.6 ± 9.1	0.387
Total protein (L), g/L	62.0 ± 9.7	57.6 ± 8.4	**0.025**
% Change in total protein	(−)5 ± 24	(−)12 ± 16	0.156
Albumin (A), g/L	34.6 ± 5.9	29.5 ± 5.8	**<0.001**
Albumin (L), g/L	29.1 ± 8.0	27.0 ± 4.5	0.074
% Change in albumin	(−)14 ± 24	(−)6 ± 21	**0.047**
ALT (A), U/L	20 ± 30	30 ± 20	**<0.001**
AST (A), U/L	37.3 ± 37.7	57.8 ± 54.2	**<0.001**
ALP (A), U/L	152 ± 119	150 ± 85	0.438
GGT (A), U/L	128 ± 115	155 ± 149	0.546
Total bilirubin (A), µmol/L	17.8 ± 21.1	24.2 ± 18.4	**0.018**
White blood cell count (A), 10^9^/L	7.73 ± 3.45	6.40 ± 3.35	**0.008**
Hemoglobin (A), g/dL	10.7 ± 1.6	10.9 ± 2.0	0.969
MCV (A), fL	86.6 ± 9.6	87.8 ± 7.8	0.504
Platelet count (A), 10^9^/L	195 ± 73	135 ± 57	**<0.001**
Platelet count (L), 10^9^/L	210 ± 104	109 ± 69	**<0.001**
% Change in platelet count	(+)12 ± 45	(−)9 ± 60	**0.002**
FIB-4 (A)	3.30 ± 1.93	6.20 ± 4.29	**<0.001**
FIB-4 (L)	4.40 ± 5.62	9.71 ± 7.98	**<0.001**
% Change in total FIB-4	(+)48 ± 162	(+)125 ± 271	**0.021**

Parameters are presented as either mean ± standard deviation; CHF, congestive heart failure; MASLD, Metabolic Dysfunction-Associated Steatotic Liver Disease; (A), at admission; (L), last follow-up visit; ALT, alanine aminotrasferase; AST, aspartate aminotrasferase; ALP, alkaline phosphatase: GGT, gamma glutamyl transpeptidase; MCV, mean corpuscular volume of red blood cells; FIB-4, fibrosis-4 index for liver fibrosis.

**Table 4:** Ascitic hematological and biochemical parameters of the major study groups at admission (A) and at the last (L) follow-up visit.

Variable	CHF n = 51	MASLD n = 47	*p*-Value
Total protein (A), g/L	36.1 ± 7.0	15.6 ± 6.1	**<0.001**
Total protein (L), g/L	29.6 ± 9.6	14.5 ± 5.9	**<0.001**
% Change in total protein	(−)18 ± 26	(−)0.3 ± 51	**0.027**
Albumin (A), g/L	18.7 ± 5.4	7.4 ± 3.7	**<0.001**
Albumin (L), g/L	14.7 ± 6.0	6.7 ± 3.1	**<0.001**
% Change in albumin	(−)18 ± 31	(−)7 ± 75	0.108
N (%) of patients with “neutrocytic ascites” (A) *	27 (52.9)	18 (38.3)	0.146

Parameters are presented either as mean ± standard deviation or n (%); CHF, congestive heart failure; MASLD, Metabolic Dysfunction-Associated Steatotic Liver Disease; (A), at admission; (L), last follow-up visit. * > than 250 neutrophils in mm^3^ in ascitic fluid.

**Table 5:** Univariate analysis of variables associated with mortality in patients with high gradient ascites.

Variable	HR (95% CI)	*p*-Value
Number of paracenteses per month of F/U	3.876 (2.799–5.367)	**<0.001**
Urea levels (A)	1.026 (1.002–1.051)	**0.032**
Creatinine levels (A)	1.003 (1.001–1.005)	**0.011**
Serum albumin levels (A)	0.969 (0.941–0.997)	**0.032**
Alanine aminotransferase levels (A)	1.007 (1.000–1.013)	**0.042**
Aspartate aminotransferase levels (A)	1.008 (1.004–0.013)	**<0.001**
Bilirubin, total levels (A)	1.015 (1.002–1.028)	**0.028**
MCV value (A)	1.041 (1.016–1.067)	**0.001**
Ascitic fluid total protein levels (A)	0.974 (0.957–0.992)	**0.005**
Ascitic fluid albumin levels (A)	0.952 (0.924–0.981)	**0.001**
FIB-4 (A)	1.059 (0.998–1.122)	0.056
FIB-4 (L)	1.045 (1.017–1.074)	**0.001**
% Change in FIB-4	1.035 (1.005–1.066)	**0.022**

95% CI, 95% confidence interval; F/U, follow-up; (A), at admission; (L), last follow-up visit; MCV, mean corpuscular volume of red blood cells; FIB-4, fibrosis-4 index for liver fibrosis.

**Table 6:** Multivariate Cox regression analysis of variables associated with mortality in patients with high grade ascites.

	Variable	Adjusted HR (95% CI)	*p*-Value
**Model #1**	Number of paracenteses per month of F/U	4.280 (2.990–6.129)	**<0.001**
	Aspartate aminotransferase levels (A)	1.009 (1.004–1.014)	**<0.001**
	MCV value (A)	1.042 (1.015–1.070)	**0.002**
	% Change in FIB-4	1.042 (1.011–1.075)	**0.008**
**Model #2**	Urea levels (A)	1.045 (1.019–1.073)	**0.001**
	Aspartate aminotransferase levels (A)	1.004 (1.000–1.009)	**0.053**
	MCV value (A)	1.027 (0.999–1.056)	**0.056**
	Ascitic fluid albumin levels (A)	0.951 (0.915–0.988)	**0.01**
	FIB-4 (L)	1.046 (1.014–1.080)	**0.005**
**Model #3**	Urea levels (A)	1.046 (1.021–1.072)	**<0.001**
	Aspartate aminotransferase levels (A)	1.005 (1.001–1.009)	**0.025**
	Ascitic fluid albumin levels (A)	0.934 (0.901–0.967)	**<0.001**

95% CI, 95% confidence interval; F/U, follow-up; (A), at admission; (L), last follow-up visit; MCV, mean corpuscular volume of red blood cells; FIB-4, fibrosis-4 index for liver fibrosis. Model #1 included all the variables that were significant at the univariate analysis of variables associated with survival. Model #2 included all the variables included in Model #1 except the number of paracenteses per month. Model #3 included only the variables that were obtained at admission.

## Data Availability

The data that support the findings of this study are available from the corresponding author upon reasonable request.

## Supplementary Materials

The following supporting information can be downloaded at: https://www.mdpi.com/article/10.3390/jcm15218537/s1, Table S1: Baseline Clinical characteristics of the 4 study subgroups; Table S2: Echocardiogram findings in the 4 study groups; Table S3: Blood hematological and biochemical parameters of the 4 study groups at admission (A) and at the last (L) follow-up visit; Table S4: Ascitic hematological and biochemical parameters of the 4 study groups at admission (A) and at the last (L) follow-up visit.

## Abbreviations

ALT, alanine aminotransferase; AST, aspartate aminotransferase; BNP, Brain Natriuretic Peptide; CHF, congestive heart failure; FIB-4, Fibrosis-4 Index; HMO, Hadassah Medical Organization; HE, hepatic encephalopathy; HRS-AKI, hepatorenal syndrome-acute kidney injury; MACE, major adverse cardiovascular events; MCV, mean corpuscular volume; MASH, metabolic dysfunction-associated steatohepatitis; MASLD, metabolic dysfunction-associated steatotic liver disease; SAAG, serum-ascites albumin gradient; SBP, spontaneous bacterial peritonitis; TP, total protein.
