# Supplementary material for: Natural History, Complications and Long-Term Prognosis of Patients with Cardiac Ascites
Source: J Clin Med. 2026 Nov 2;15(21):8537. doi: 10.3390/jcm15218537 (PMC13764308; doi:10.3390/jcm15218537)
Supplement: Supplementary file 1 [file jcm-15-08537-s001.zip › jcm-4592282-supplementary.pdf]

Supplementary Tables

Table S1: Baseline Clinical characteristics of the 4 study subgroups

**Variable**	**CHF only** n=40	**CHF and MASLD** n=11	**MASLD only** n=36	**MASLD and Cardiac Pathology** n=11	**p-value**
Age at presentation (years)	66.4 ± 14.9	67.3 ± 5.7	66. 7 ± 11.2	72.0 ± 8.2	0.549
Follow-up period (months)	21.6 ± 17.9	28.7 ± 32.0	17. 9 ± 13.4	13.0 ± 15.7	0.164
Number of paracenteses during F/U	8.30 ± 8.02	11.36 ± 8.53	9.50 ± 6.66	8.64 ± 11.32	0.287
Number of paracenteses per month of F/U	0.56 ± 0.48	0.89 ± 0.96	0.88 ± 0.68	1.5 ± 1.3	0.068
Chronic lung disease	6 (15.0)	8 (72.7)	10 (27.8)	4 (36.4)	**0.003**
Ischemic heart disease	26 (65.0)	7 (63.6)	12 (33.3)	6 (54.5)	**0.039**
Chronic atrial fibrillation	26 (65.0)	6 (54.5)	6 (16.7)	6 (54.5)	**<0.001**
Underwent a liver biopsy	1 (2.5)	2 (18.2)	18 (50.0)	1 (9.1)	**<0.001**
Presence of esophageal varices	0 (0.0)	4 (36.4)	23 (63.9)	3 (27.3)	**<0.001**
Presence of hepatic encephalopathy	2 (5.0)	4 (36.4)	20 (55.6)	2 (18.2)	**<0.001**
Presence of splenomegaly	2 (5.0)	8 (72.7)	22 (61.1)	4 (36.4)	**<0.001**
Albumin infusion during paracentesis	6 (15.0)	3 (27.3)	13 (36.1)	5 (45.5)	0.083

Parameters are presented either as mean ± standard deviation or as n (%). CHF, congestive heart failure; MASLD, Metabolic Dysfunction-Associated Steatotic Liver Disease; F/U, follow-up.

Table S2: Echocardiogram findings in the 4 study groups					
**Variable**	**CHF only**	**CHF and MASLD**	**MASLD only**	**MASLD and Cardiac pathology**	**p-value**
**Mitral regurgitation**	n=37	n=10	n=35	n=10	**<0.001**
No finding	4 (10.8)	5 (50)	24 (68.6)	4 (40)	
Mild	8 (21.6)	1 (10)	8 (22.9)	1(10)	
Moderate	14 (37.8)	3 (30)	2 (5.7)	3 (30)	
Severe	11 (29.7)	1 (10)	1 (2.9)	2 (20)	
**Tricuspid regurgitation**	n=36	n=10	n=36	n=10	**<0.001**
No finding	2 (5.6)	5 (50)	22 (61)	2 (20)	
Mild	5 (13.9)	2 (20)	11 (30.6)	3 (30)	
Moderate	10 (27.8)	1 (10)	2 (5.6)	2 (20)	
Severe	19 (52.8)	2 (20)	1 (2.8)	3 (30)	
**Pulmonary hypertension**	n=34	n=10	n=36	n=11	**<0.001**
No finding	4 (11.8)	6 (60)	32 (88.9)	5 (45.5)	
Mild	6 (17.6)	0 (0.0)	3 (8.3)	0 (0.0)	
Moderate	11 (32.4)	2 (20)	0 (0.0)	4 (36.4)	
Severe	13 (38.2)	2 (20)	1 (2.8)	2 (18.2)	
**Left Ventricular Dysfunction**	n=39	n=11	n=36	n=11	**<0.001**
No finding	3 (7.7)	4 (36.4)	35 (97.2)	1 (9.1)	
Mild	9 (23.1)	2 (18.2)	0 (0.0)	6 (54.5)	
Moderate	11 (28.2)	4 (36.4)	1 (2.8)	1 (9.1)	
Severe	16 (41.0)	1 (9.1)	0 (0.0)	3 (27.3)	
**Right Ventricular Dysfunction**	n=38	n=10	n=35	n=8	**<0.001**
No finding	2 (5.3)	5 (50)	35 (100)	1 (12.5)	
Mild	6 (15.8)	1 (10)	0 (0.0)	5 (62.5)	
Moderate	14 (36.8)	2 (20)	0 (0.0)	1 (12.5)	
Severe	16 (42.1)	2 (20)	0 (0.0)	1 (12.5)	
Parameters are presented in number (%) of patients that underwent echocardiography examination and a result of the parameter examined was reported. CHF, congestive heart failure; MASLD, Metabolic Dysfunction-Associated Steatotic Liver Disease.					

Table S3: Blood hematological and biochemical parameters of the 4 study groups at admission (A) and at the last (L) follow up visit					
**Variable**	**CHF only** n=40	**CHF and MASLD** n=11	**MASLD only** n=36	**MASLD and Cardiac pathology** n=11	**p-value**
Urea (A), mmol/L	18.3. ± 10.2	15.6 ± 7.3	7.6 ± 5.0	16.1 ± 10.5	**<0.001**
Creatinine (A), μmol/L	182 ± 116	230 ± 161	102 ± 58	194 ± 180	**<0.001**
Creatinine (L), (μmol/L)	236 ± 126	387 ± 170	251 ± 115	306 ± 196	0.06
% Change in creatinine	57 ± 95	118 ± 147	175 ± 133	184 ± 221	**0.001**
Total protein (A), g/L	68.5 ± 10.3	64.6 ± 13.4	68.5 ± 8.1	60.5 ± 10.1	0.169
Total protein (L), g/L	61.9 ± 9.6	62.7 ± 10.8	57.9 ± 9.0	56.9 ± 6.3	0.162
% Change in total protein	(-) 7 ± 22	(-) 4 ± 32	(-) 15 ± 13	(-) 4 ± 19	0.138
Albumin (A), g/L	35.3 ± 4.6	31.9 ± 8.8	29.7 ± 6.4	28.8 ± 3.2	**<0.001**
Albumin (L), g/L	28.5 ± 7.3	31.3 ± 10.3	26.9 ± 4.5	27.0 ± 4.6	0.286
% Change in albumin	(-) 18 ± 21	(-) 0.17 ± 29	(-) 6 ± 22	(-) 6 ± 17	0.054
ALT (A), (U/L)	21.4 ± 34.0	14.5 ± 8.9	27.6 ± 15.7	36.3 ± 31.3	**<0.001**
AST (A), (U/L)	36.8 ± 40.5	39.4 ± 26.8	52.6 ± 31.9	74.7 ± 97.8	**0.001**
Total bilirubin (A), μmol/L	19.5 ± 23.5	11.7 ± 4.2	25.7 ± 19.1	19.3 ±15.5	**0.02**
MCV (A), fL	87.0 ± 9.2	85.4 ± 11.7	88.0 ± 8.5	87.3 ± 5.5	0.854
Platelet count (A), 10^9/L	191 ± 70	208 ± 85	124 ± 53	172 ± 56	**<0.001**
FIB-4 (A)	3.29 ± 2.12	3.37 ± 1.01	6.66 ± 4.41	4.68 ± 3.65	**<0.001**
FIB-4 (L)	3.44 ± 3.95	7.92 ± 8.93	10.08 ± 8.81	8.49 ± 4.32	**<0.001**
% Change in FIB-4	29 ± 149	119 ± 192	109 ± 270	178 ± 282	**0.007**

Parameters are presented as mean ± standard deviation; CHF, congestive heart failure; MASLD, Metabolic Dysfunction-Associated Steatotic Liver Disease; (A), at admission; (L), last follow- up visit; ALT, alanine aminotrasferase; AST, aspartate aminotransferase; MCV, mean corpuscular volume; FIB-4, fibrosis- 4 index for liver fibrosis.

Table S4: Ascitic biochemical parameters of the 4 study groups at admission (A) and at the last (L) follow up visit					
**Variable**	**CHF only** n=40	**CHF and MASLD** n=11	**MASLD only** n=36	**MASLD and Cardiac pathology** n=11	**p-value**
Total protein (A), g/L	35.6 ± 6.3	37.5 ± 9.2	15.6 ± 6.6	15.8 ± 4.2	**<0.001**
Total protein (L), g/L	28.8 ± 9.6	32.7 ± 9.6	15.1 ± 5.8	12.3 ± 6.1	**<0.001**
% Change in total protein	(-) 19 ± 27	(-) 14 ± 25	(-) 9 ± 54	(-) 29 ± 26	**0.008**
Albumin (A), g/L	18.5 ± 4.9	19.4 ± 7.0	7.3 ± 4.0	7.45 ± 2.7	**<0.001**
Albumin (L), g/L	14.3 ± 5.8	16.4 ± 6.4	7.0 ± 3.2	5.73 ± 2.7	**<0.001**
% Change in albumin	(-) 20 ± 31	(-) 11 ± 33	(-) 16 ± 82	(-) 22 ± 25	0.229

Parameters are presented as mean ± standard deviation; CHF, congestive heart failure; MASLD, Metabolic Dysfunction-Associated Steatotic Liver Disease; (A), at admission; (L), last follow- up visit.
